# Supplementary material for: A transcriptomic based deconvolution framework for assessing differentiation stages and drug responses of AML
Source: NPJ Precis Oncol. 2024 May 18;8:105. doi: 10.1038/s41698-024-00596-9 (PMC11102519; doi:10.1038/s41698-024-00596-9)
Supplement: Supplementary file 2 — Supplementary Figures [file 41698_2024_596_MOESM2_ESM.pdf]

Figure S1

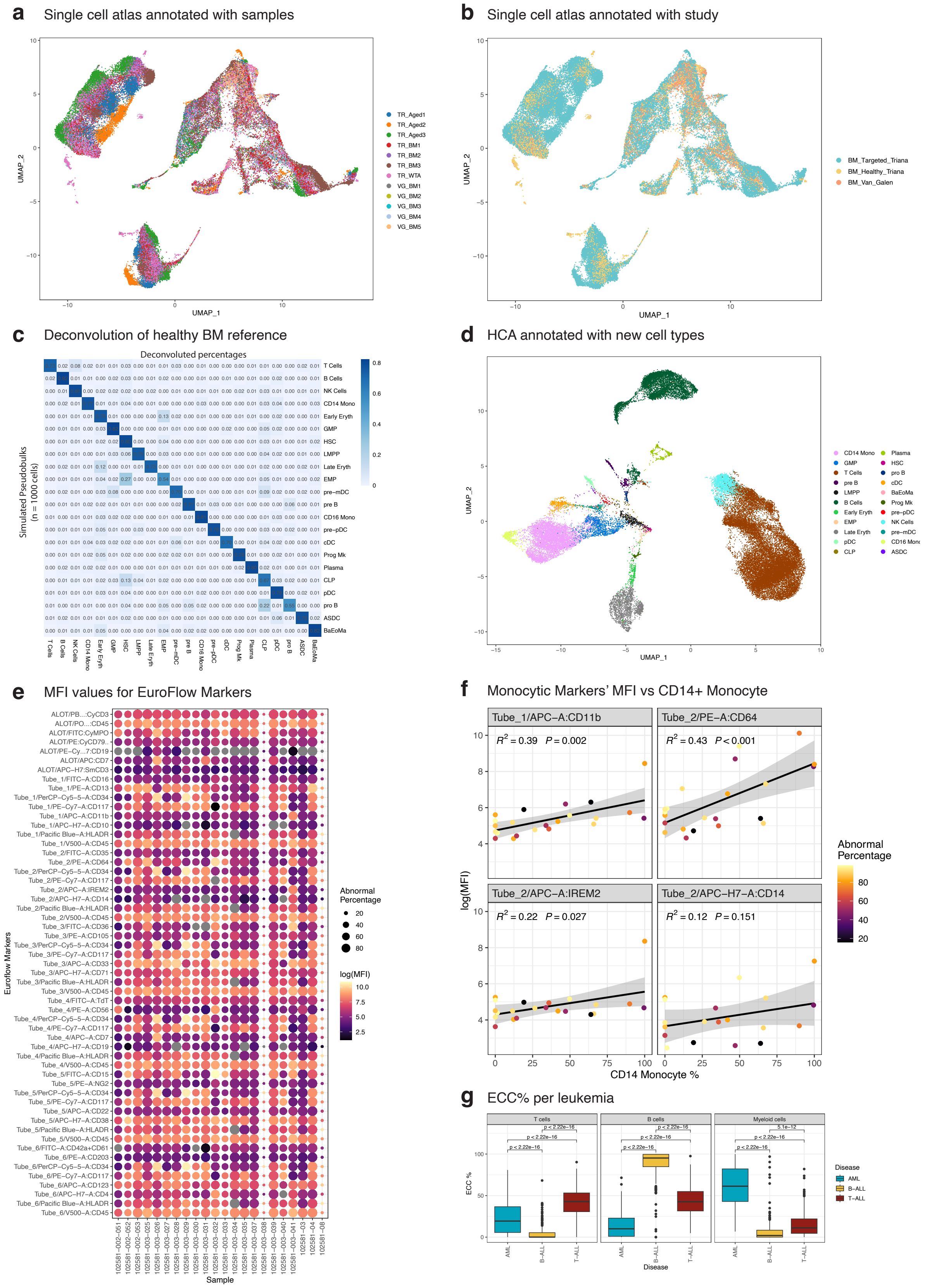
a Single cell atlas annotated with samples
UMAP_1
UMAP_2
TR_Aged1
TR_Aged2
TR_Aged3
TR_BM1
TR_BM2
TR_BM3
TR_WTA
VG_BM1
VG_BM2
VG_BM3
VG_BM4
VG_BM5
b Single cell atlas annotated with study
BM_Targeted_Triana
BM_Healthy_Triana
BM_Van_Galen
c Deconvolution of healthy BM reference
Deconvoluted percentages
Simulated Pseudobulks (n = 1000 cells)
T Cells
B Cells
NK Cells
CD14 Mono
Early Eryth
GMP
HSC
LMPP
Late Eryth
EMP
pre−mDC
pre B
CD16 Mono
pre−pDC
cDC
Prog Mk
Plasma
CLP
pDC
pro B
ASDC
BaEoMa
d HCA annotated with new cell types
CD14 Mono
GMP
T Cells
pre B
LMPP
B Cells
Early Eryth
EMP
Late Eryth
pDC
CLP
Plasma
HSC
pro B
cDC
BaEoMa
Prog Mk
pre−pDC
NK Cells
pre−mDC
CD16 Mono
ASDC
e MFI values for EuroFlow Markers
Euroflow Markers
Sample
Abnormal Percentage
log(MFI)
f Monocytic Markers' MFI vs CD14+ Monocyte
Tube_1/APC−A:CD11b
$R^2 = 0.39$ $P = 0.002$
Tube_2/PE−A:CD64
$R^2 = 0.43$ $P < 0.001$
Tube_2/APC−A:IREM2
$R^2 = 0.22$ $P = 0.027$
Tube_2/APC−H7−A:CD14
$R^2 = 0.12$ $P = 0.151$
log(MFI)
CD14 Monocyte %
Abnormal Percentage
g ECC% per leukemia
T cells
B cells
Myeloid cells
ECC %
Disease
AML
B−ALL
T−ALL

Figure S1 – Single-cell integration evaluation & flow markers

A Healthy BM reference overlaid on UMAP separated by samples **B** and by different studies. **C** Similar to Figure 1D; heatmap showing the deconvolution results of simulated pseudobulks from healthy BM reference via an over-abundant cell type (80%). **D** UMAP for HCA subset with the cell type annotations, which were lifted via Azimuth framework. **E** Dot plot of MFI values of all EuroFlow markers for all samples, size of the dot represents the abnormal cell percentages per sample assigned with EuroFlow analyses. **F** MFI values of four EuroFlow monocytic markers (CD11b, CD64, IREM2 and CD14) and CD14+ Monocyte %. Colors indicate the abnormal percentage of cells by flow cytometry assignments. **G** Boxplot plot showing the ECCs of TARGET AML (n=187) and ALL cohorts (n=267 for B-ALL, n=265 for T-ALL), colors indicate the primary diagnosis of each leukemic sample (Supplementary Table S1).

Figure S2

a Patient meta summary from all studies

Characteristic	Overall, N = 1,267[1]	BEAT, N = 412[1]	LEUCE, N = 452[1]	LUMC, N = 96[1]	TARGET, N = 156[1]	TCGA, N = 151[1]
Age	55 (0, 89)	62 (2, 87)	57 (17, 87)	NA (Inf, -Inf)	9 (0, 23)	56 (22, 89)
Unknown	97	1	0	96	0	0
Sex						
female	567/1,267 (45%)	183/412 (44%)	195/452 (43%)	44/96 (46%)	77/156 (49%)	68/151 (45%)
male	700/1,267 (55%)	229/412 (56%)	257/452 (57%)	52/96 (54%)	79/156 (51%)	83/151 (55%)
FAB						
M0	51/761 (6.7%)	5/90 (5.6%)	27/381 (7.1%)	0/0 (NA%)	4/140 (2.9%)	15/150 (10%)
M1	189/761 (25%)	7/90 (7.8%)	129/381 (34%)	0/0 (NA%)	17/140 (12%)	36/150 (24%)
M2	138/761 (18%)	10/90 (11%)	54/381 (14%)	0/0 (NA%)	37/140 (26%)	37/150 (25%)
M3	56/761 (7.4%)	11/90 (12%)	30/381 (7.9%)	0/0 (NA%)	0/140 (0%)	15/150 (10%)
M4	155/761 (20%)	24/90 (27%)	59/381 (15%)	0/0 (NA%)	43/140 (31%)	29/150 (19%)
M5	145/761 (19%)	31/90 (34%)	69/381 (18%)	0/0 (NA%)	30/140 (21%)	15/150 (10%)
M6	14/761 (1.8%)	0/90 (0%)	10/381 (2.6%)	0/0 (NA%)	2/140 (1.4%)	2/150 (1.3%)
M7	13/761 (1.7%)	2/90 (2.2%)	3/381 (0.8%)	0/0 (NA%)	7/140 (5.0%)	1/150 (0.7%)
Unknown	506	322	71	96	16	1
ELN						
Adverse	197/686 (29%)	148/390 (38%)	0/0 (NA%)	0/0 (NA%)	9/148 (6.1%)	40/148 (27%)
Favorable	203/686 (30%)	107/390 (27%)	0/0 (NA%)	0/0 (NA%)	64/148 (43%)	32/148 (22%)
Intermediate	286/686 (42%)	135/390 (35%)	0/0 (NA%)	0/0 (NA%)	75/148 (51%)	76/148 (51%)
Unknown	581	22	452	96	8	3
Blast	72 (0, 100)	60 (0, 98)	77 (1, 99)	75 (15, 99)	74 (14, 100)	71 (0, 100)
Unknown	144	109	31	0	4	0
Primary Diagnosis						
Acute myeloid leukemia with inv(3)(q21q26.2) or t(3;3)(q21;q26.2); RPN1-EVI1	17/1,267 (1.3%)	8/412 (1.9%)	7/452 (1.5%)	2/96 (2.1%)	0/156 (0%)	0/151 (0%)
Acute myeloid leukemia with mutated CEBPA	37/1,267 (2.9%)	28/412 (6.8%)	0/452 (0%)	0/96 (0%)	9/156 (5.8%)	0/151 (0%)
Acute myeloid leukemia with mutated NPM1	163/1,267 (13%)	96/412 (23%)	0/452 (0%)	27/96 (28%)	7/156 (4.5%)	33/151 (22%)
Acute myeloid leukemia with myelodysplasia-related changes	213/1,267 (17%)	88/412 (21%)	114/452 (25%)	11/96 (11%)	0/156 (0%)	0/151 (0%)
Acute myeloid leukemia with t(10;11)(p12;q23.3); MLLT10-KMT2A	9/1,267 (0.7%)	0/412 (0%)	0/452 (0%)	0/96 (0%)	9/156 (5.8%)	0/151 (0%)
Acute myeloid leukemia with t(6;11)(q27;q23.3); MLLT4-KMT2A	5/1,267 (0.4%)	0/412 (0%)	0/452 (0%)	0/96 (0%)	5/156 (3.2%)	0/151 (0%)
Acute myeloid leukemia with t(6;9)(p23;q34); DEK-NUP214	8/1,267 (0.6%)	3/412 (0.7%)	3/452 (0.7%)	1/96 (1.0%)	1/156 (0.6%)	0/151 (0%)
Acute myeloid leukemia with t(8;21)(q22;q22); RUNX1-RUNX1T1	60/1,267 (4.7%)	9/412 (2.2%)	20/452 (4.4%)	3/96 (3.1%)	21/156 (13%)	7/151 (4.6%)
Acute myeloid leukemia with t(9;11)(p22;q23); MLLT3-MLL	35/1,267 (2.8%)	9/412 (2.2%)	8/452 (1.8%)	4/96 (4.2%)	12/156 (7.7%)	2/151 (1.3%)
Acute myeloid leukemia, CBF-beta/MYH11	101/1,267 (8.0%)	26/412 (6.3%)	29/452 (6.4%)	10/96 (10%)	28/156 (18%)	8/151 (5.3%)
Acute myeloid leukemia, NOS	492/1,267 (39%)	91/412 (22%)	215/452 (48%)	35/96 (36%)	64/156 (41%)	87/151 (58%)
Acute promyelocytic leukaemia, PML-RAR-alpha	62/1,267 (4.9%)	17/412 (4.1%)	29/452 (6.4%)	2/96 (2.1%)	0/156 (0%)	14/151 (9.3%)
Myeloid sarcoma	3/1,267 (0.2%)	3/412 (0.7%)	0/452 (0%)	0/96 (0%)	0/156 (0%)	0/151 (0%)
Therapy related myeloid neoplasm	62/1,267 (4.9%)	34/412 (8.3%)	27/452 (6.0%)	1/96 (1.0%)	0/156 (0%)	0/151 (0%)

[1] Median (Range) or Frequency (%)

b AML studies on ternary plot

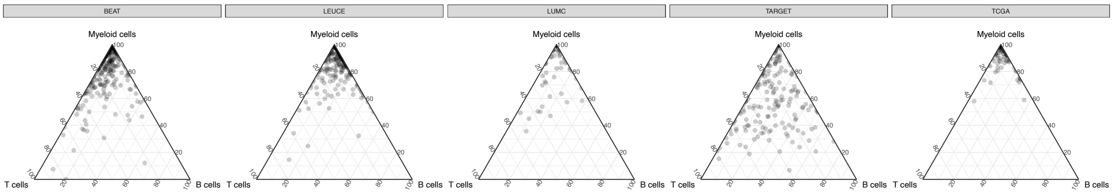

Figure S2 – Overview of AML cohorts

A Detailed table for sample characteristics for age, sex, FAB, ELN, and primary diagnosis (WHO) for each cohort. Missing data is labeled as 'Unknown' for each attribute. *AML, NOS patients represents %39 of WHO classes. **B** Ternary plots like Figure 1G for all five AML cohorts.

Figure S3

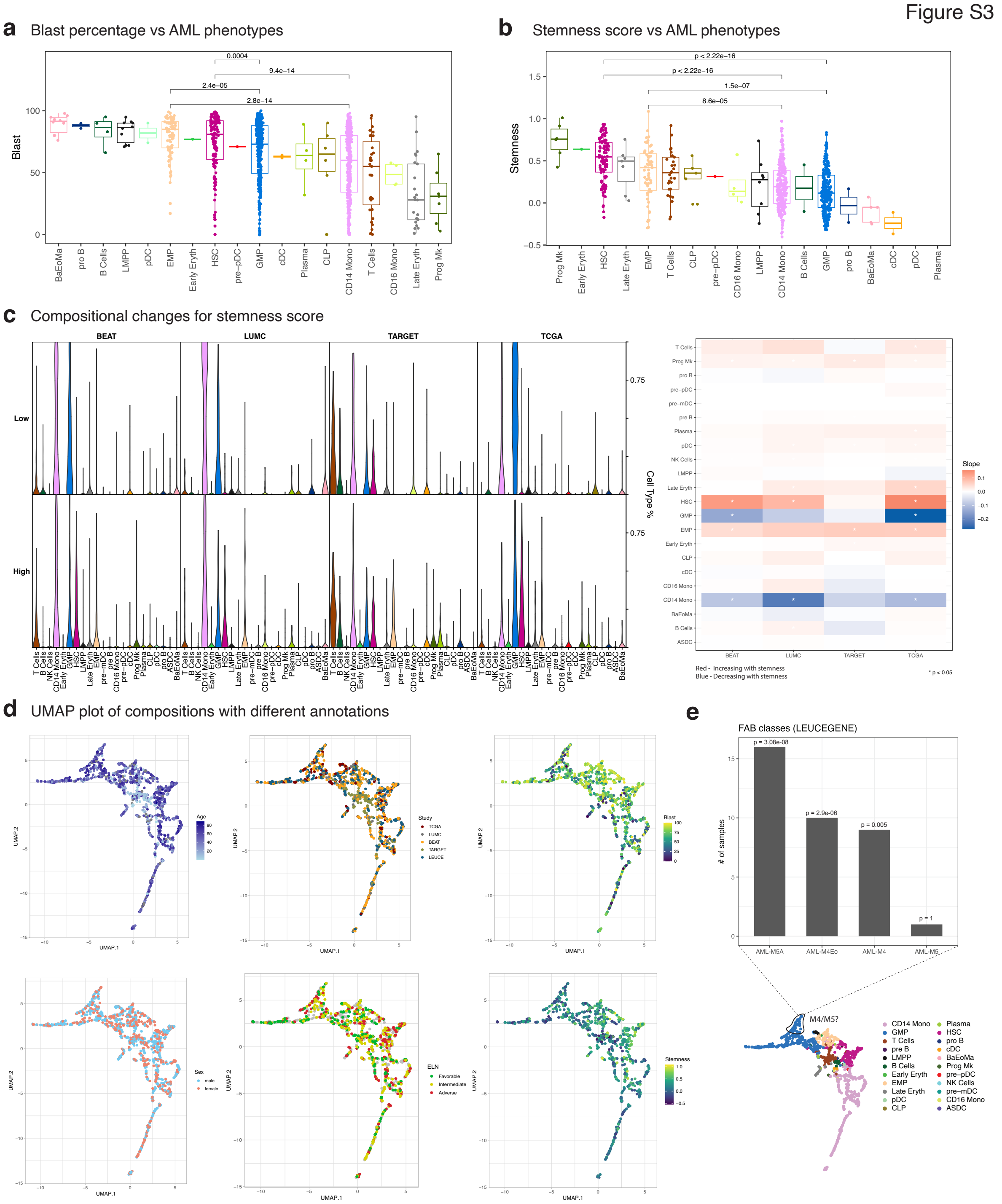
a Blast percentage vs AML phenotypes
b Stemness score vs AML phenotypes
c Compositional changes for stemness score
d UMAP plot of compositions with different annotations
e
FAB classes (LEUCEGENE)
Red - Increasing with stemness
Blue - Decreasing with stemness
M4/M5?

Figure S3 – Stemness score and meta data on UMAP

A Box plots of blast percentage vs AML phenotype and **B** Stemness score vs AML phenotype. Boxplot were sorted according to median values. **C** Compositional changes of each cohort (except LEUCEGENE as it lacks entire list of genes to calculate stemness score) for low and high stemness score (split by median). Violin plots show the deconvoluted cellular percentages for 22 cell type and heatmap on the right panel summarizes the changes. A linear model is fit to calculate the slope and significance per cell type vs. stemness, and a red slope indicates more of that cell type with higher stemness score, and blue is the vice versa. Asterix indicates a statistically significant trend ($P < 0.05$). **D** UMAP plots of deconvoluted percentages (ECCs) annotated via age, study, reported blast level, sex, ELN and stemness score. **E** Bar plot of extended FAB classifications of LEUCEGENE patients with GMP assignment and M4/5 morphology

Figure S4

a Drug resistance predictions for all drugs

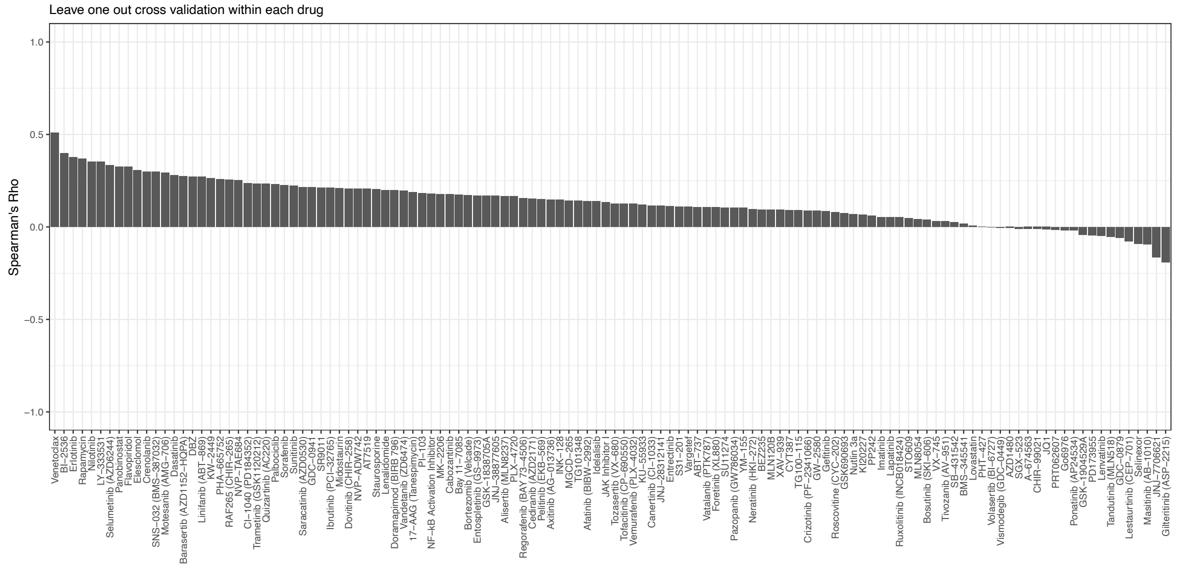

b Drug resistance predictions stratified for primary diagnosis

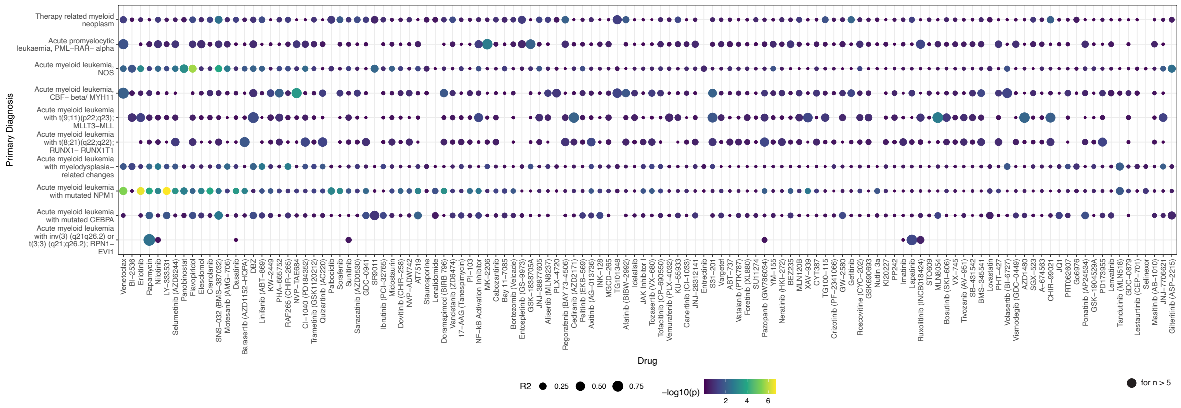

Figure S4 – Summary plots for *ex-vivo* drug response predictions

A Spearman ρ values for the predictions of the drug resistance of 122 small molecule inhibitors from BEAT-AML (see Supplementary Table S7). For each drug, the resistance per sample is predicted via random forest algorithm using ECCs at leave-one-out cross validation setting. **B** Dot plot showing the stratified associations with predicted resistance values. Colors of the dot show the significance and sizes indicate the correlation strength. Groups with more than 5 samples were included in the plot.

Figure S5

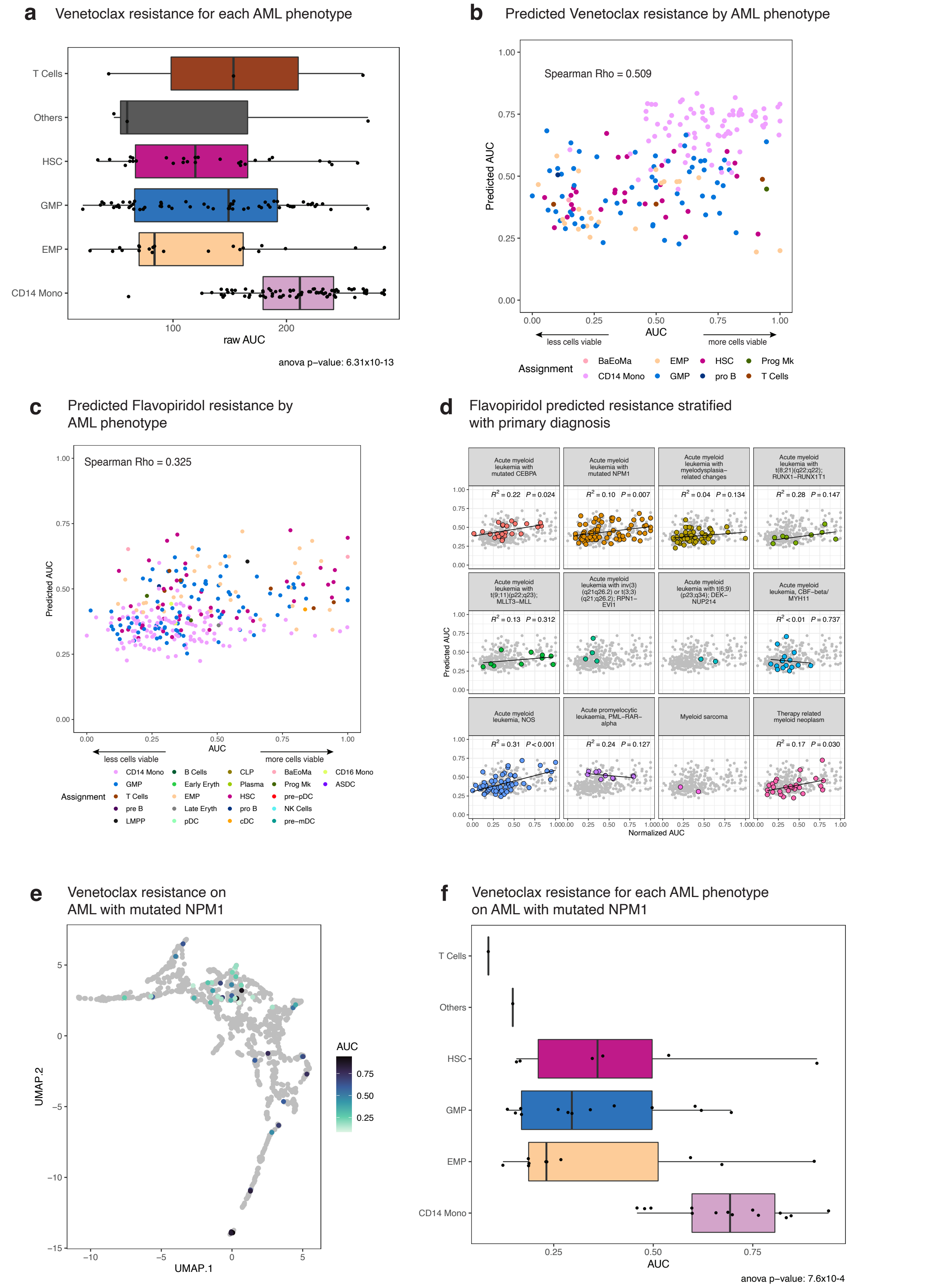
a
Venetoclax resistance for each AML phenotype
T Cells
Others
HSC
GMP
EMP
CD14 Mono
100
200
raw AUC
anova p−value: 6.31x10-13
b
Predicted Venetoclax resistance by AML phenotype
Spearman Rho = 0.509
Predicted AUC
AUC
less cells viable
more cells viable
Assignment
BaEoMa
EMP
HSC
Prog Mk
CD14 Mono
GMP
pro B
T Cells
c
Predicted Flavopiridol resistance by AML phenotype
Spearman Rho = 0.325
Predicted AUC
AUC
less cells viable
more cells viable
Assignment
CD14 Mono
GMP
T Cells
pre B
LMPP
B Cells
Early Eryth
EMP
Late Eryth
pDC
CLP
Plasma
HSC
pro B
cDC
BaEoMa
Prog Mk
pre−pDC
NK Cells
pre−mDC
CD16 Mono
ASDC
d
Flavopiridol predicted resistance stratified with primary diagnosis
Acute myeloid leukemia with mutated CEBPA
Acute myeloid leukemia with mutated NPM1
Acute myeloid leukemia with myelodysplasia−related changes
Acute myeloid leukemia with t(8;21)(q22;q22); RUNX1−RUNX1T1
Acute myeloid leukemia with t(9;11)(p22;q23); MLLT3−MLL
Acute myeloid leukemia with inv(3)(q21q26.2) or t(3;3)(q21;q26.2); RPN1−EVI1
Acute myeloid leukemia with t(6;9)(p23;q34); DEK−NUP214
Acute myeloid leukemia, CBF−beta/MYH11
Acute myeloid leukemia, NOS
Acute promyelocytic leukaemia, PML−RAR−alpha
Myeloid sarcoma
Therapy related myeloid neoplasm
Predicted AUC
Normalized AUC
e
Venetoclax resistance on AML with mutated NPM1
UMAP.2
UMAP.1
AUC
f
Venetoclax resistance for each AML phenotype on AML with mutated NPM1
T Cells
Others
HSC
GMP
EMP
CD14 Mono
AUC
anova p−value: 7.6x10-4

Figure S5 – Venetoclax response on different WHO classes

A Venetoclax resistance split by AML phenotypes. All drugs' one-way anova p values are provided in Supplementary Table S10. **B** Venetoclax resistance predictions with random forest annotated with patients' AML phenotype. **C** Flavopiridol resistance predictions with random forest annotated with patients' AML phenotype. **D** Flavopiridol resistance predictions are faceted and highlighted by WHO classes, classes with >5 samples were fit linear models thus R^2 and p values are shown. **E** UMAP plot showing Venetoclax resistance only for NPM1 mutated samples **F** Venetoclax resistance split by AML phenotype only for NPM1 mutated samples.

Figure S6

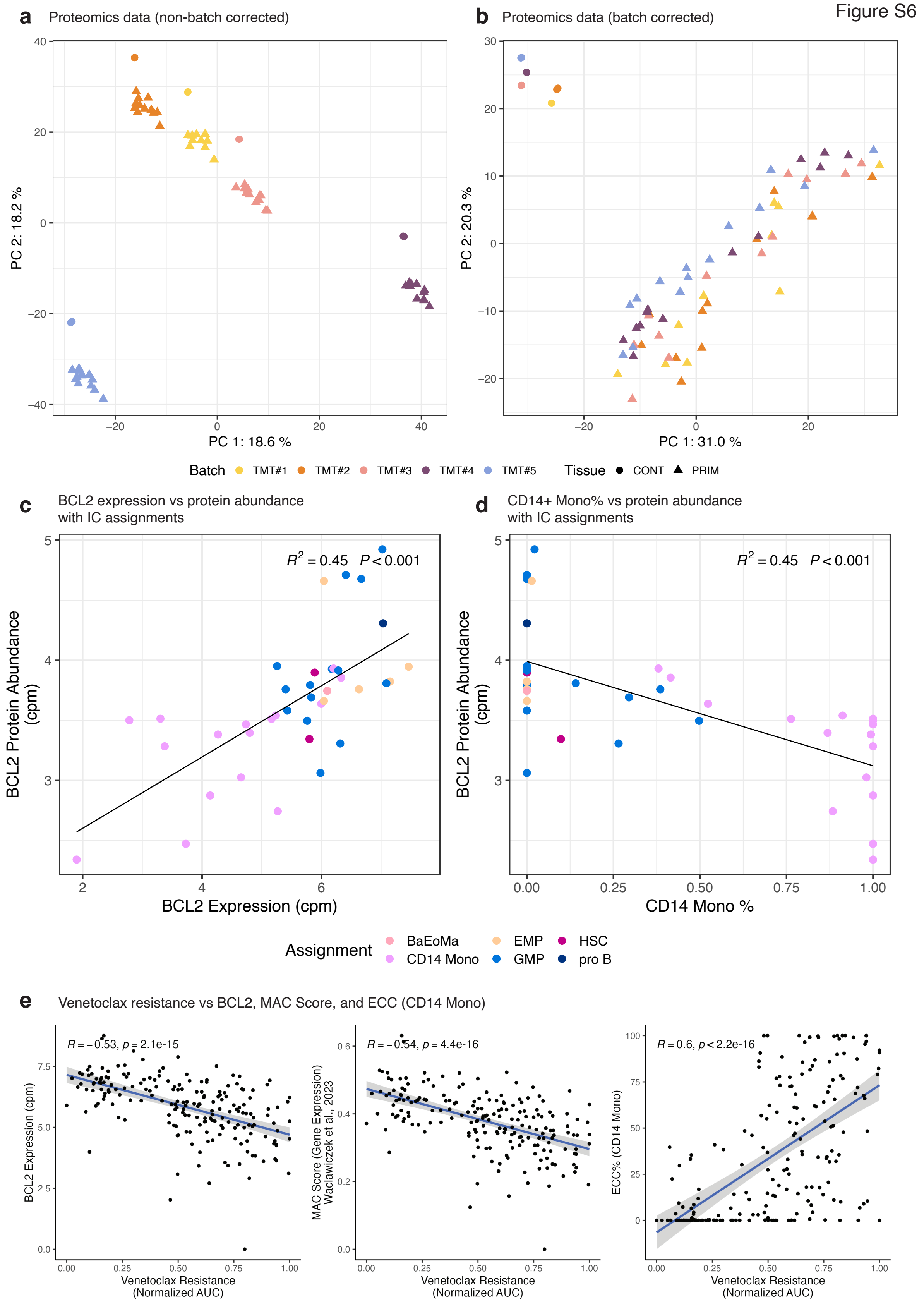
a Proteomics data (non-batch corrected)
PC 2: 18.2 %
PC 1: 18.6 %
b Proteomics data (batch corrected)
PC 2: 20.3 %
PC 1: 31.0 %
Batch TMT#1 TMT#2 TMT#3 TMT#4 TMT#5
Tissue CONT PRIM
c BCL2 expression vs protein abundance with IC assignments
R2 = 0.45 P < 0.001
BCL2 Protein Abundance (cpm)
BCL2 Expression (cpm)
d CD14+ Mono% vs protein abundance with IC assignments
R2 = 0.45 P < 0.001
BCL2 Protein Abundance (cpm)
CD14 Mono %
Assignment BaEoMa CD14 Mono EMP GMP HSC pro B
e Venetoclax resistance vs BCL2, MAC Score, and ECC (CD14 Mono)
R = −0.53, p = 2.1e-15
BCL2 Expression (cpm)
Venetoclax Resistance (Normalized AUC)
R = −0.54, p = 4.4e-16
MAC Score (Gene Expression) Waclawiczek et al., 2023
Venetoclax Resistance (Normalized AUC)
R = 0.6, p < 2.2e-16
ECC% (CD14 Mono)
Venetoclax Resistance (Normalized AUC)

Figure S6 – QC plots & BCL-2 expression for proteomics

A PCA plot of proteomics data (primary AML n = 39) without batch correction with TMT controls and **B** same samples with batch correction. All samples are log-cpm normalized. **C** *BCL-2* expression vs protein abundance annotated with patients' AML phenotypes ($R^2 = 0.45$, $P < 0.001$). **D** CD14+ Monocytes percentage vs BCL-2 protein abundance annotated with patients' AML phenotypes ($R^2 = 0.45$, $P < 0.001$). **E** Venetoclax resistance in *ex-vivo* compared to: BCL2 gene, MAC Score, BCL2/(BCL-xL+MCL1) calculated with corresponding gene signatures, and the estimated CD14 Mono percentages.

Figure S7

a Flow gating strategy on Euroflow Panel

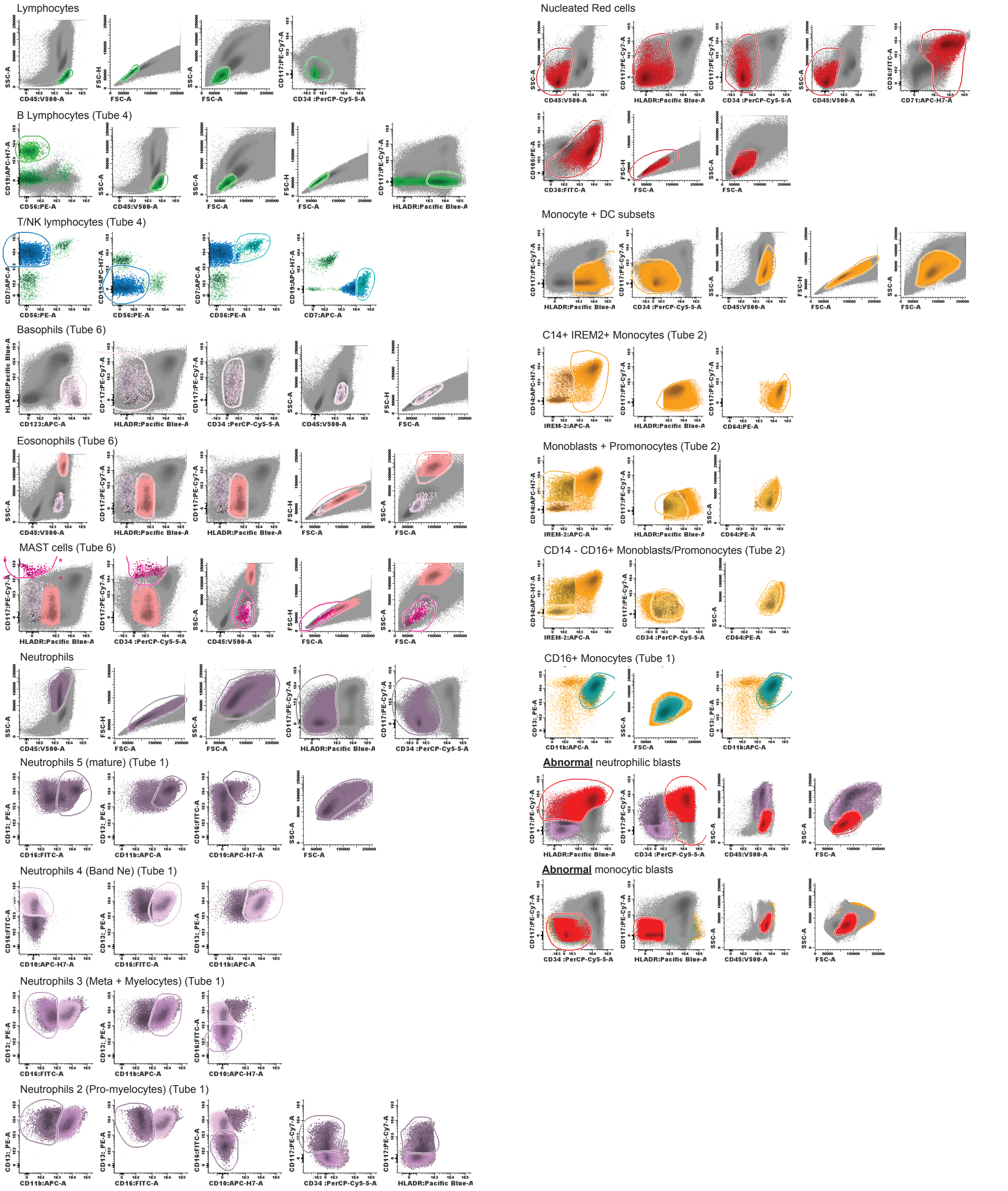

Figure S7 – Flow gating strategy for EuroFlow panels

a An example of EuroFlow gating strategy on one of our flow samples. Seven tubes (ALOT orientation tube + 6 AML assignment tubes) were used per patient to sort normal-like cell (lymphocytes, eosonophils, neutrophils etc.) and abnormal cell subsets (abnormal neutrophilic blast, abnormal monocytic blast). Different colors indicate related cell (sub)types.

Supplementary Tables

Tab 1 TARGET deconvolutions

Deconvolutions of TARGET AML and ALL cohorts

Tab 2 Deconvolution Results

Estimated cell compositions for 22 cell types along with meta data of 1,350 samples

Tab 3 EuroFlow MFI values

EuroFlow MFI values of abnormal populations of LUMC samples

Tab 4 EuroFlow cell type percentages

EuroFlow gating results for different cell types (abnormals are not annotated) of LUMC samples

Tab 5 BEAT AUC

Min-max normalized AUC values for 122 small-molecule inhibitors from BEAT-AML

Tab 6 BEAT AUC RF Predictions

Predictions of each drug resistance with random forest at LOOCV

Tab 7 Drug Spearman Rho

Spearman rho values for drug resistance predictions of 122 small-molecule inhibitors

Tab 8 Drug Associations stratified with WHO

Predicted drug and normalized resistance associations within each primary diagnosis

Tab 9 Anova - Drug vs Cell Types (All)

One-way anova test results with sample phenotype assignments for drug resistances

Tab 10 Anova - Drug vs Cell Types (NPM1)

One-way anova test results with sample phenotype assignments for drug resistances (NPM1 only)

Tab 11 Univariate Venetoclax

The results of linear model associations of Venetoclax resistance to different attributes

Tab 12 Multivariate Venetoclax

The results of linear model association of Venetoclax resistance with different attributes

Tab 13 Deconvolution of Ven/Aza patients

Estimated cell compositions for 22 cell types along with meta data of BEAT-AML Ven/Aza samples

Tab 14 Related Proteomics Counts

Normalized proteomics counts used for NPM1 samples
